# Mutations in the floral regulator gene *HUA2* restore flowering to the Arabidopsis *trehalose 6-phosphate synthase1* (*tps1*) mutant

**DOI:** 10.1093/plphys/kiaf225

**Published:** 2025-06-05

**Authors:** Liping Zeng, Vasiliki Zacharaki, Sam W van Es, Yanwei Wang, Markus Schmid

**Affiliations:** Beijing Advanced Innovation Centre for Tree Breeding by Molecular Design, College of Biological Sciences and Biotechnology, Beijing Forestry University, Beijing 100083, People's Republic of China; School of Grassland Science, Beijing Forestry University, Beijing 100083, People's Republic of China; Umeå Plant Science Centre, Department of Plant Physiology, Umeå University, Umeå SE-901 87, Sweden; Department of Plant Biology, Linnean Center for Plant Biology, Swedish University of Agricultural Sciences, Uppsala S-75007, Sweden; Beijing Advanced Innovation Centre for Tree Breeding by Molecular Design, College of Biological Sciences and Biotechnology, Beijing Forestry University, Beijing 100083, People's Republic of China; Beijing Advanced Innovation Centre for Tree Breeding by Molecular Design, College of Biological Sciences and Biotechnology, Beijing Forestry University, Beijing 100083, People's Republic of China; Umeå Plant Science Centre, Department of Plant Physiology, Umeå University, Umeå SE-901 87, Sweden; Department of Plant Biology, Linnean Center for Plant Biology, Swedish University of Agricultural Sciences, Uppsala S-75007, Sweden

## Abstract

Plant growth and development are regulated by many factors, including carbohydrate availability and signaling. Trehalose 6-phosphate (T6P), which is synthesized by TREHALOSE-6-PHOSPHATE SYNTHASE 1 (TPS1), is positively associated with and functions as a signal that informs the cell about the carbohydrate status. Mutations in *TPS1* negatively affect the growth and development of Arabidopsis (*Arabidopsis thaliana*), and complete loss-of-function alleles are embryo-lethal, which can be overcome using inducible expression of *TPS1* (*GVG::TPS1*) during embryogenesis. Using ethyl methane sulfonate mutagenesis in combination with genome re-sequencing, we have identified several alleles in the floral regulator gene *HUA2* that restore flowering in *tps1-2 GVG::TPS1*. Genetic analyses using an *HUA2* T-DNA insertion allele, *hua2-4*, confirmed this finding. RNA-seq analyses demonstrated that *hua2-4* has widespread effects on the *tps1-2 GVG::TPS1* transcriptome, including key genes and pathways involved in regulating flowering. Higher order mutants combining *tps1-2 GVG::TPS1* and *hua2-4* with alleles in the key flowering time regulators *FLOWERING LOCUS T* (*FT*), *SUPPRESSOR OF OVEREXPRESSION OF CONSTANS 1* (*SOC1*), and *FLOWERING LOCUS C* (*FLC*) were constructed to analyze the role of *HUA2* during floral transition in *tps1-2* in more detail. Our findings demonstrate that loss of *HUA2* can restore flowering in *tps1-2 GVG::TPS1*, in part through activation of *FT*, with contributions from the upstream regulators *SOC1* and *FLC*. Interestingly, we found that mutation of *FLC* is sufficient to induce flowering in *tps1-2 GVG::TPS1*. Furthermore, we observed that mutations in *HUA2* modulate carbohydrate signaling and that this regulation might contribute to flowering in *hua2-4 tps1-2 GVG::TPS1*.

## Teaser

*HUA2* represents an important genetic link between energy management and flowering time regulation by modulating T6P-dependent carbohydrate signaling and activating the floral integrator *FLOWER LOCUS T*.

## Introduction

Plants have evolved intricate signaling mechanisms that enable them to monitor a wide range of environmental and endogenous cues and adjust their physiology, growth, and development accordingly. Adjustments occur more or less constantly, but developmental phase transitions such as germination, the switch from juvenile to adult growth, or the induction of flowering and reproductive development are under particularly stringent control.

In Arabidopsis (*Arabidopsis thaliana*), the floral transition is controlled by environmental factors including exposure to prolonged periods of cold (vernalization), ambient temperature, day length (photoperiod), light quality, and endogenous signals such as plant age, diverse hormones including gibberellic acid, and carbohydrate signaling (Srikanth and Schmid 2011; Romera-Branchat et al. 2014; Cho et al. 2017). Eventually, these signaling pathways converge on and regulate the expression of key floral integrator genes such as *FLOWERING LOCUS T* (*FT*) and *SUPPRESSOR OF OVEREXPRESSION OF CONSTANS 1* (*SOC1*) (Kardailsky et al. 1999; Moon et al. 2005; Kobayashi and Weigel 2007; Turck et al. 2008; Lee and Lee 2010; Jung et al. 2012). *FT* is induced in response to permissive photoperiod in the leaf vasculature where it is also translated. The FT protein is then transported via the phloem to the shoot apical meristem (SAM) where it interacts with the bZIP transcription factor FD and 14-3-3 proteins to form the florigen activation complex (Abe et al. 2005; Wigge et al. 2005; Mathieu et al. 2007; Taoka et al. 2011; Collani et al. 2019). In contrast, *SOC1* is induced and acts largely at the SAM, both downstream and in parallel to *FT* (Yoo et al. 2005; Lee and Lee 2010). Eventually, these factors induce flower meristem identity genes such as *LEAFY* and *APETALA1* at the SAM, thus completing the floral transition (Weigel and Nilsson 1995; Liljegren et al. 1999; Blázquez and Weigel 2000).

Apart from photoperiod, carbohydrate signaling has been shown to be necessary for *FT* expression (Wahl et al. 2013). Sucrose is the major product of photosynthesis and the most common transport sugar. However, rather than measuring sucrose concentration directly, plants employ trehalose 6-phosphate (T6P) as a readout and signal of sucrose availability (Goddijn and van Dun 1999; Lunn et al. 2006; Martins et al. 2013; Yadav et al. 2014; Figueroa and Lunn 2016). T6P is the intermediate of trehalose synthesis. It is synthesized from glucose 6-phosphate and uridine diphosphate glucose by TREHALOSE 6-PHOSPHATE SYNTHASE (TPS) and subsequently dephosphorylated by TREHALOSE 6-PHOSPHATE PHOSPHATASE (TPP) (Cabib and Leloir 1958).

In Arabidopsis, there are 11 *TPS* genes (*AtTPS1–AtTPS11*), which can be divided into 2 subclades, class I and class II, and 10 TPP genes (*TPPA–TPPJ*) (Leyman et al. 2001; Lunn 2007; Vandesteene et al. 2012). Among the class I *TPS* genes (*AtTPS1–AtTPS4*), only *AtTPS1*, *AtTPS2*, and *AtTPS4* exhibit demonstrable catalytic activity, while *AtTPS3* contains a premature translational stop codon and is likely a pseudogene (Blázquez et al. 1998; Van Dijck et al. 2002; Lunn 2007; Delorge et al. 2015). Class II *TPS* genes (*AtTPS5–AtTPS11*), for which no TPS activity has been detected, have been reported to play roles in cell size regulation, thermotolerance, and resistance to cold and salt stress. However, the underlying molecular mechanisms remain largely unclear (Chary et al. 2008; Ramon et al. 2009; Singh et al. 2011; Tian et al. 2019; Van Leene et al. 2022). The main T6P synthase in Arabidopsis is TPS1. *TPS1* loss-of-function mutations are embryonic lethal (Eastmond et al. 2002), but homozygous *tps1-2* mutants could be established by dexamethasone-inducible expression of *TPS1* (*GVG::TPS1*) during embryogenesis (van Dijken et al. 2004). Interestingly, the resulting homozygous *tps1-2 GVG:TPS1* plants did not flower unless treated with dexamethasone (van Dijken et al. 2004). At the molecular level, late flowering of *tps1-2 GVG::TPS1* has been attributed to the combined misregulation of key flowering time genes. In particular, *tps1-2 GVG::TPS1* mutant plants fail to induce *FT* in leaves even under permissive photoperiod. In addition, *MIR156* and its targets, the *SQUAMOSA PROMOTER BINDING PROTEIN LIKE* genes, which together constitute the age pathway, are also misregulated in *tps1-2 GVG::TPS1* (Wahl et al. 2013). More recently, T6P in conjunction with nitrogen signaling has been implicated in the regulation of the floral repressor *FLOWERING LOCUS C* (*FLC*) (Gramma et al. 2024). The authors also reported very late flowering of the uninduced *tps1-2 GVG::TPS1* line, which is different from the original report (van Dijken et al. 2004) and our own observations (Zacharaki et al. 2022). The differences in flowering of *tps1-2 GVG::TPS1* observed by different groups are most likely caused subtle and difficult to control differences in growth conditions, such as soil and light quality, temperature fluctuations, etc., which are well known to modulate TPS1/T6P and its downstream target SnRK1 (Nunes et al. 2013; Frank et al. 2018; Hwang et al. 2019; Reichelt et al. 2023; Gramma et al. 2024). Nevertheless, many questions regarding the regulation of plant growth and development by the T6P pathway remain open.

In an EMS suppressor screen, we have recently reported dozens of mutations that partially restored flowering and seed set in *tps1-2 GVG::TPS1*, including several alleles in *SNF1 KINASE HOMOLOG 10* (*KIN10*) and *HOMOLOG OF YEAST SUCROSE NONFERMENTING 4* (*SNF4*), 2 subunits of Arabidopsis SNF1-Related Kinase 1 (SnRK1) (Jung et al. 2012; Zacharaki et al. 2022), an evolutionarily conserved regulator of cellular energy homeostasis that acts antagonistically with the target of rapamycin (TOR) pathway (Margalha et al. 2019; Artins and Caldana 2022; Ingargiola et al. 2023; Artins et al. 2024).

Here, we identified several new alleles in *HUA2* (*At5g23150*) that partially rescue the *tps1-2 GVG::TPS1* phenotype. Mutations in *HUA2* were originally identified in a genetic screen as enhancers of the *AGAMOUS* (*AG*) allele *ag-4* (Chen and Meyerowitz 1999). In addition, *HUA2* has also been reported to affect shoot morphology and function as a repressor of flowering (Doyle et al. 2005; Wang et al. 2007). At the molecular level, HUA2 has been suggested to function as a putative transcription factor but has also been implicated in RNA processing (Cheng et al. 2003). We show that 3 different EMS-induced point mutations in *HUA2* restore flowering in *tps1-2 GVG::TPS1* and verify this finding using a previously described T-DNA insertion allele, *hua2-4*. RNA-seq analyses revealed widespread effects of *hua2-4* on the *tps1 GVG::TPS1* transcriptome, including activation of flower integrator genes such as *SOC1* and *AGAMOUS-LIKE 24* (*AGL24*). Genetic analyses demonstrated that induction of flowering in *tps1-2 GVG::TPS1* required functional *FT*. Furthermore, we observed that loss of *FLC* is sufficient to induce flowering in *tps1-2 GVG::TPS1*. Interestingly, *hua2-4* also attenuated the induction of known SnRK1 target genes in response to carbon starvation. Taken together, our results identify mutations in *HUA2* as suppressors of the non-flowering phenotype of *tps1-2 GVG::TPS1* and provide insights into the underlying genetic and molecular pathways.

## Results

### Mutations in *hua2* restore flowering in *tps1-2 GVG::TPS1*

To identify previously undescribed components of the T6P pathway, we recently conducted a suppressor screen in which the non-flowering *tps1-2 GVG::TPS1* mutant was subjected to ethyl methane sulfonate (EMS) mutagenesis. In total, 106 M2 mutant plants in which flowering and seed set were at least partially restored were isolated, and EMS-induced SNPs were identified by whole genome sequencing in a subset of 65 mutants (Zacharaki et al. 2022). To identify additional candidate suppressor genes in which SNPs were overrepresented, we expanded this list to 92 mutants by sequencing the genomes of another 27 mutant lines (Supplementary Table S1).

**Figure 1.**
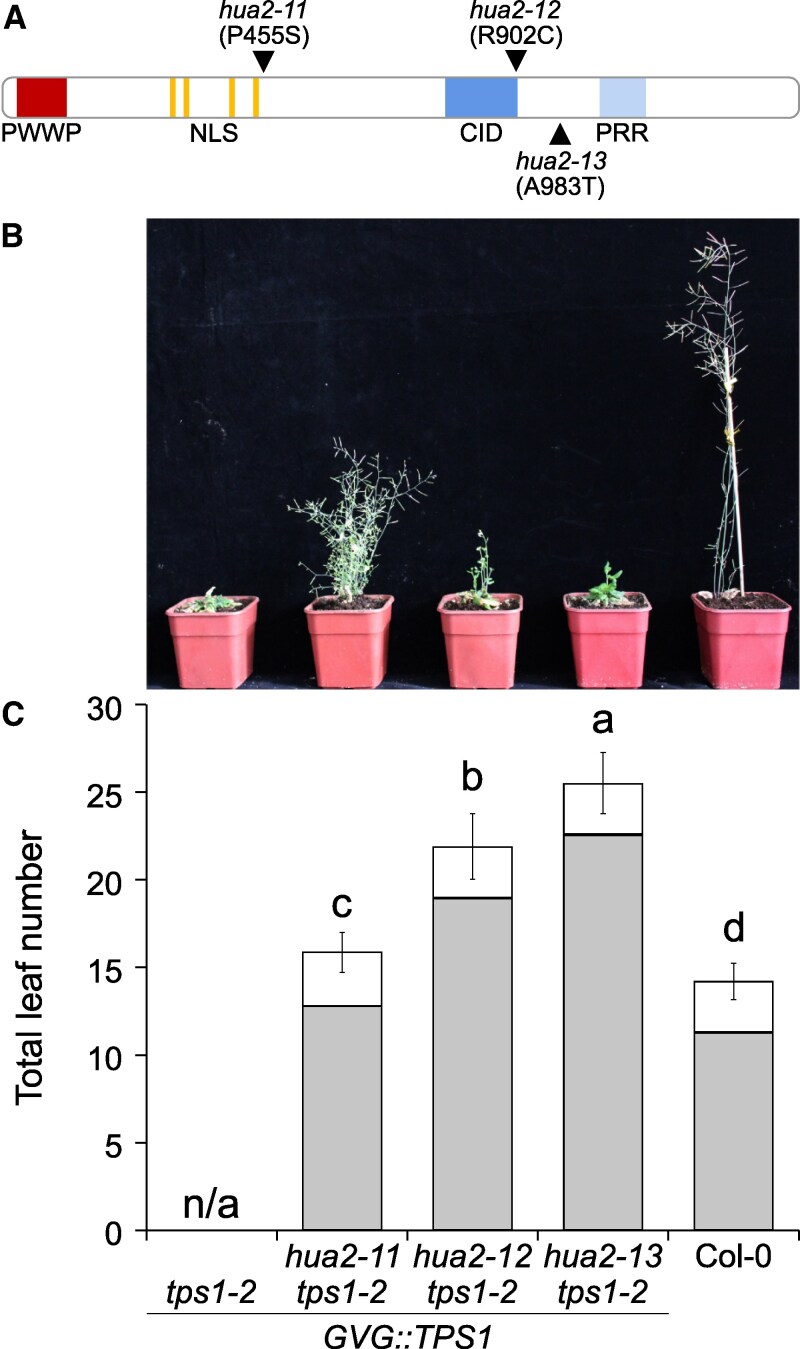
EMS-induced mutations in *HUA2* induce flowering in *tps1-2 GVG::TPS1* background. **A)** Schematic drawing of HUA2 indicating the position and the amino acid changes caused by the ethyl methanesulfonate (EMS)-induced mutations *hua2-11* (P455S), *hua2-12* (R902C), and *hua2-13* (A983T). PWWP: PWWP protein domain; NLS: nuclear localization signal; CID: RNA polymerase II (RNAPII) C-terminal domain (CTD) interaction domain; PRR: proline-rich region. **B)** Phenotype of 9-wk-old *tps1-2 GVG::TPS1*, *hua2-11 tps1-2 GVG::TPS1, hua2-12 tps1-2 GVG::TPS1,* and *hua2-13 tps1-2 GVG::TPS1* and wild-type Col-0 plants grown in LD with a photoperiod of 16 h light at 22 °C and 8 h darkness at 20 °C. *GVG::TPS1* designates a dexamethasone-inducible *TPS1* transgene present in the genotype. **C)** Flowering time of genotypes is given as total leaf number (rosette leaves: gray; cauline leaves: white) determined after bolting. Error bars represent the standard deviation of the total leaf number based on 20 individuals per genotype (Supplementary Table S4). ANOVA Tukey's multiple comparisons test was applied, and letters represent the statistical differences among genotypes (*P*  *<*  *0.001*).

Analysis of these 92 genome sequences for genes with multiple independent EMS-induced mutations identified 3 SNPs in the coding sequence of *HUA2* (*AT5G23150*) (Supplementary Tables S2 and S3). The 3 alleles result in non-synonymous amino acid substitutions, namely A983T, P455S, and R902C. We refer to these new EMS-induced suppressor lines as *hua2-11* (line #8-1-1), *hua2-12* (line #233-14-1), and *hua2-13* (line #164-9-1), respectively (Fig. 1A). The polymorphism R902C resides at the C-terminal end of the HUA2 CID motif (RNA Pol-II C-terminal domain [CTD] interaction domain). The *hua2-11* (line #8-1-1) allele was also detected in 2 additional suppressor lines, #57-2-1 and #30-34 (Supplementary Tables S2 and S3). As these 3 lines share most EMS-induced SNPs genome-wide, we assume these lines originate from the same parental plant.

Importantly, flowering was restored in all 3 *hua2* alleles, even though all 3 mutant lines produced substantially more leaves before making the transition to flowering than Col-0 control plants (Fig. 1, B and C). The flowering time of *hua2-11* was 32.15 d, whereas *hua2-12* and *hua2-13* flowered after 46.5 and 50.9 d, respectively, compared with Col-0, which flowered after 25.2 d (Supplementary Table S4, experiment 1). Thus, the 3 mutants form an allelic series with *hua2-11* being the strongest and *hua2-13* being the weakest allele. As *HUA2* has previously been implicated in flowering time regulation and has been shown to regulate the expression of a group of MADS-box transcription factors known to form a floral repressive complex in Arabidopsis (Doyle et al. 2005; Wang et al. 2007; Lee et al. 2013; Posé et al. 2013; Jali et al. 2014; Yan et al. 2016), we considered mutations in this gene as likely to be causal for the restoration of flowering in the *tps1-2 GVG::TPS1* suppressor lines.

**Figure 2.**
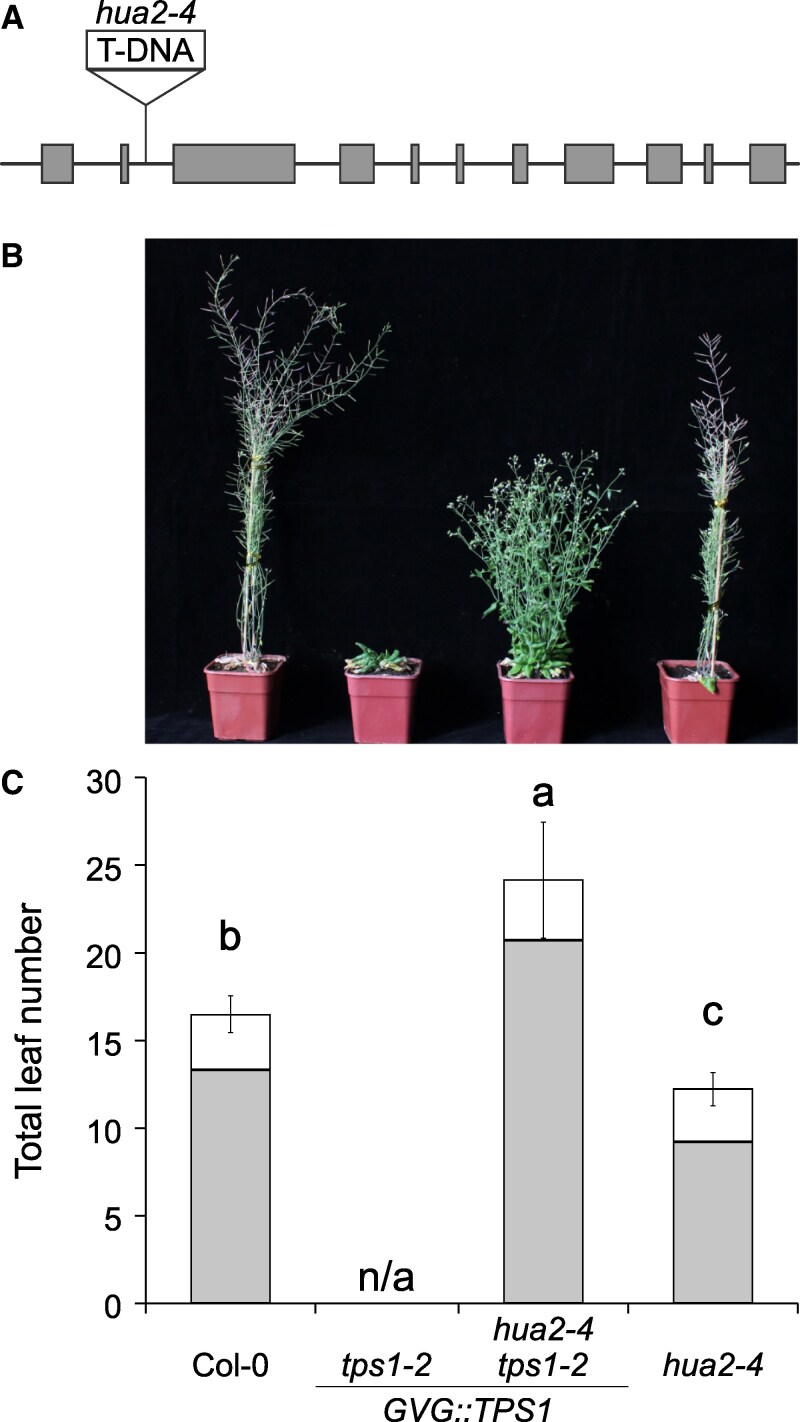
A T-DNA insertion in *HUA2* partially rescues the flowering time phenotype of *tps1-2 GVG::TPS1*. **A)** Schematic drawing of the *HUA2* locus indicating the position of the T-DNA insertion (SALK_032281C) in the 2nd intron in *hua2-4*. Gray boxes indicated exons. **B**, **C)** Phenotypic analysis **(B)** and flowering time **(C)** of 9-wk-old wild-type Col-0, *tps1-2 GVG::TPS1*, *hua2-4 tps1-2 GVG::TPS1* and *hua2-4* plants grown in LD with a photoperiod of 16 h light at 22 °C and 8 h darkness at 20 °C. *GVG::TPS1* designates a dexamethasone-inducible *TPS1* transgene present in the genotype. Flowering time was scored as total leaf number (rosette leaves: gray; cauline leaves: white) after bolting. Error bars represent the standard deviation of the total leaf number based on 20 individuals per genotype (Supplementary Table S4). ANOVA Tukey's multiple comparisons test was applied, and letters represent the statistical differences among genotypes (*P*  *<*  *0.001*).

Since the 3 *hua2* alleles described above were generated through EMS mutagenesis, it is possible that other independent mutations not linked to *HUA2* could be involved in partially rescuing the *tps1-2 GVG::TPS1* phenotype. To confirm that mutations in *HUA2* are causal for the suppression of the *tps1-2* non-flowering phenotype, we crossed *tps1-2 GVG::TPS1* with *hua2-4*, a previously described *hua2* loss-of-function mutant that carries a T-DNA insertion in the 2nd intron (Fig. 2A) (Doyle et al. 2005). Of the F2 plants homozygous for the *tps1-2* mutations, only those approx. 25% that were homozygous for the *hua2-4* T-DNA insertion flowered without application of dexamethasone. Similar to *hua2-11 tps1-2 GVG::TPS1* (Fig. 1, B and C), *hua2-4 tps1-2 GVG::TPS1* double mutants displayed a bushy shoot phenotype and were moderately late flowering (Fig. 2, B and C; Supplementary Table S4, experiment 2). Importantly, *TPS1* expression was not altered in *hua2-4 tps1-2 GVG::TPS1* when compared with *tps1-2 GVG::TPS1* (Supplementary Fig. S1), indicating that the effect of *hua2* mutations on flowering in *tps1-2* was not caused by inadvertent activation of the *GVG::TPS1* transgene. Taken together, our findings confirm that recessive mutations in *HUA2* are responsible for the induction of flowering in *tps1-2 GVG::TPS1*. Our findings also suggest that *HUA2* normally functions by repressing flowering either directly or indirectly through the promotion of floral repressors.

### *hua2-4* has widespread effects on the *tps1-2 GVG::TPS1* transcriptome

To identify possible downstream targets of *HUA2* whose misexpression might explain the induction of flowering in the suppressor mutant, we performed RNA-seq analysis in leaves of 21-d-old *tps1-2 GVG::TPS1* plants, *tps1-2 GVG::TPS1* plants treated with dexamethasone, and the *hua2-4 tps1-2 GVG::TPS1* double mutant. Plants were grown under long days (LD) (16 h light, 8 h dark) in the presence or absence of dexamethasone and samples were collected at Zeitgeber time 4 (ZT4, 4 h after lights on) as expression of *TPS1* peaks early in the morning (Redmond et al. 2025). Genes that were differentially expressed in 3 independent replicates per genotype and treatment were identified using Cuffdiff 2 (Trapnell et al. 2012).

**Figure 3.**
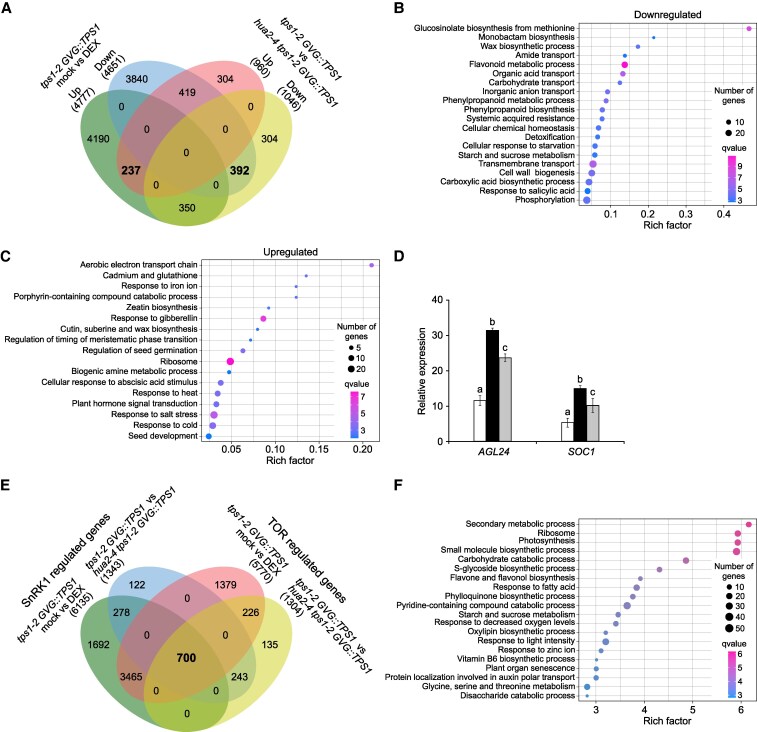
Characterization of the *hua2-4 tps1-2 GVG::TPS1* transcriptome. **A)** 4-way Venn diagram of genes that are differentially expressed in *tps1-2 GVG::TPS1* in response to dexamethasone (DEX) treatment and/or differentially expressed in *hua2-4 tps1-2 GVG::TPS1* when compared with *tps1-2 GVG::TPS1*. *GVG::TPS1* designates a dexamethasone-inducible *TPS1* transgene present in the genotype. Expression estimates and lists of DEGs were calculated based on 3 biological RNA-seq replicates per genotype. **B)** GO analysis of 392 genes downregulated in *tps1-2 GVG::TPS1* in response to dexamethasone treatment and in *hua2-4 tps1-2 GVG::TPS1*. **C)** GO analysis of 237 genes upregulated in *tps1-2 GVG::TPS1* in response to dexamethasone treatment and in *hua2-4 tps1-2 GVG::TPS1.*  **D)** Relative expression of *AGAMOUS-LIKE 24* (*AGL24*) and *SUPPRESSOR OF OVEREXPRESSION OF CONSTANS 1* (*SOC1*) in *tps1-2 GVG::TPS1* (white), *tps1-2 GVG::TPS1* treated with dexamethasone (black), and *hua2-4 tps1-2 GVG::TPS1* (gray). *AGL24* and *SOC1* are significantly differentially expressed. Error bars indicate the standard deviation based on 3 biological replicates. ANOVA Tukey's multiple comparisons test was applied, and letters represent the statistical differences among genotypes (*P*  *<*  *0.001*). **E)** 4-way Venn diagram of genes known sucrose non-fermenting 1 (SNF1)-related protein kinases (SnRK1) and target of rapamycin (TOR) target genes that are differentially expressed in *tps1-2 GVG::TPS1* in response to dexamethasone treatment and/or differentially expressed in *hua2-4 tps1-2 GVG::TPS1* when compared with *tps1-2 GVG::TPS1*. **F)** GO analysis of 700 SnRK1 and TOR target genes differentially expressed in *tps1-2 GVG::TPS1* in response to dexamethasone application and loss of *HUA2* function.

We observed that dexamethasone treatment significantly affected the expression of 9,428 genes in *tps1-2 GVG::TPS1*. Of these, 4,777 and 4,651 genes were upregulated and downregulated, respectively (Fig. 3A). In contrast, mutation of *hua2* affected the expression of only 2,006 genes, of which 960 and 1,046 genes were upregulated and downregulated in *hua2-4 tps1-2 GVG::TPS1*, respectively (Fig. 3A). In total, our RNA-seq analysis identified 1,398 genes that are differentially expressed in *tps1-2 GVG::TPS1* in response to dexamethasone application and the *hua2-4* mutation. Importantly, *HUA2* expression is not changed in *tps1-2 GVG::TPS1* in response to dexamethasone application, suggesting that *hua2* might induce flowering largely by activating a pathway not normally regulated by the T6P pathway (Supplementary Fig. S2).

Since both dexamethasone application and mutations in *hua2* can induce flowering in *tps1-2 GVG::TPS1,* we next searched for genes that were repressed or induced in response to either treatment. We identified 392 genes that were downregulated in *tps1-2 GVG::TPS1* in response to dexamethasone application and mutations in *hua2* (Fig. 3A), which is significantly more than expected by chance (Fisher's exact test; *P* = 3.05 × 10^−22^). Gene ontology (GO) analysis revealed that among others, processes such as flavonoid metabolism (GO:0009812), carbohydrate transport (GO:0008643), and starvation response (GO:0009267) were significantly enriched, which is in line with the well-established role of *TPS1* in remodeling carbohydrate metabolism (Fig. 3B; Supplementary Tables S5 and S6).

In addition, we identified 237 genes that were induced in response to dexamethasone and in *hua2-4 tps1-2 GVG::TPS1*, which is significantly more than expected by chance (Fisher's exact test; *P* = 7.33 × 10^−9^). Among these genes, GO categories related to the response to gibberellin (GO:0009739) and the regulation of timing of meristematic phase transition (GO:0048506) are of particular interest as they are directly linked to the transition to flowering (Fig. 3C; Supplementary Tables S6 and S7). Importantly, among the genes induced in *tps1-2 GVG::TPS1* by dexamethasone and *hua2* were *SOC1* and *AGL24*, 2 MADS-domain transcription factors known to promote the transition to flowering (Fig. 3D; Supplementary Table S8). In contrast, other known flowering time regulators such as *CONSTANS* (*CO*), *FT*, and *TWIN SISTER OF FT* (*TSF*) are either hardly detectable (Supplementary Fig. S3A), possibly because of the collection time of the RNA-seq samples at ZT4, or did not change significantly in *hua2* and in response to dexamethasone treatment (Supplementary Fig. S3B). In summary, our transcriptome analysis identified several downstream genes and pathways whose misregulation could contribute to the induction of flowering in *tps1-2 GVG::TPS1* in response to dexamethasone application or loss of *hua2* (Supplementary Fig. S3; Supplementary Table S8).

Next, we wanted to test if *hua2* induced flowering directly by activating genes such as *SOC1* and *AGL24* (Fig. 3D), or if *hua2* might at least in part act through the canonical SnRK1 and TOR energy signaling pathways. We found that 6,135 and 1,343 of the genes previously shown to be regulated by SnRK1 (Pedrotti et al. 2018) were differentially expressed in *tps1-2 GVG::TPS1* in response to dexamethasone application and loss of *HUA2*, respectively (Fig. 3E). Similarly, we detected 5,770 and 1,304 genes in our data set that have previously been shown to be regulated by TOR (Fig. 3E) (Xiong et al. 2013; Fu et al. 2021). In total, we identified 700 known TOR and SnRK1 target genes that were also misregulated in *tps1-2 GVG::TPS1* in response to dexamethasone application and loss of *HUA2* function (Fig. 3E; Supplementary Table S9), significantly more than expected by chance (Fisher's exact test; *P* = 3.36 × 10^−148^).

Interestingly, these 700 genes were enriched for GO categories central to SnRK1 and TOR signaling such as ribosomes, photosynthesis, and energy metabolism (carbohydrate metabolic process; starch and sucrose metabolism; disaccharide catabolic process) (Fig. 3F). Taken together, these analyses suggest that *hua2-4* might not simply act as a bypass mutation but regulates flowering in *tps1-2 GVG::TPS1* partially by modulating energy signaling.

### Induction of flowering of *tps1-2 GVG::TPS1* by *hua2-4* requires *FT*

**Figure 4.**
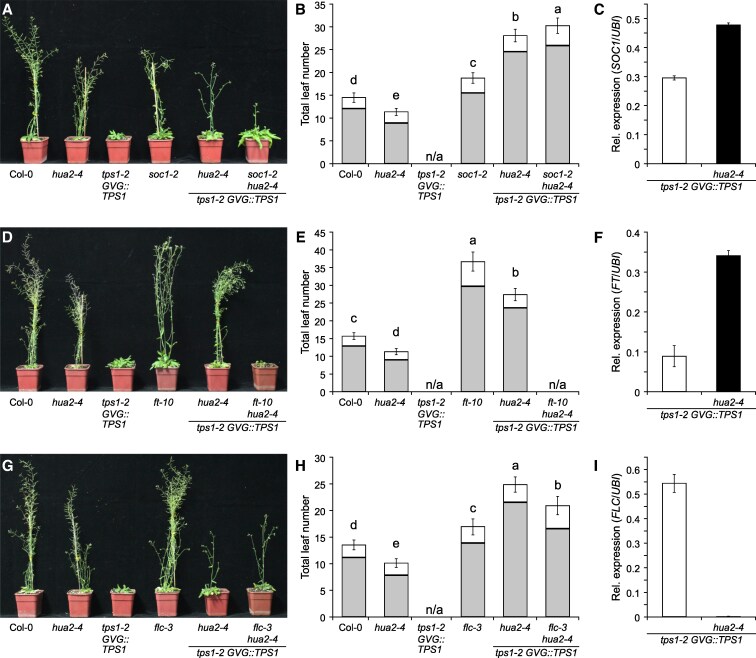
Genetic interactions between *tps1-2*, *hua2-4*, and floral regulators *SOC1*, *FT*, and *FLC*. **A**, **B)** Phenotypes **(A)** and flowering time **(B)** of Col-0, *hua2-4*, *tps1-2 GVG::TPS1*, and *soc1-2* mutant combinations. **D, E)** Phenotypes **(D)** and flowering time **(E)** of Col-0, *hua2-4*, *tps1-2 GVG::TPS1*, and *ft-10* mutant combinations. **G**, **H)** Phenotypes **(G)** and flowering time **(H)** of Col-0, *hua2-4*, *tps1-2 GVG::TPS1*, and *flc-3* mutant combinations. Flowering time **(B**, **E**, **H)** was scored as total leaf number (rosette leaves: gray; cauline leaves: white) after bolting. *GVG::TPS1* designates a dexamethasone-inducible *TPS1* transgene present in the genotype. Error bars represent the standard deviation of the total leaf number based on 20 individuals per genotype, except *ft-10* for which 10 individuals were phenotyped (Supplementary Table S4). ANOVA Tukey's multiple comparisons test was applied, and letters represent the statistical differences among genotypes (*P*  *<*  *0.001*). **C**, **F**, **I)** Relative expression of *SUPPRESSOR OF OVEREXPRESSION OF CONSTANS 1* (*SOC1*) **(C)**, *FLOWERING LOCUS T* (*FT*) **(F)**, and *FLOWERING LOCUS C* (*FLC*) **(I)** in *tps1-2 GVG::TPS1* and *hua2-4 tps1-2 GVG::TPS1*. Gene expression was determined by RT-qPCR at the end of the LD (zeitgeber [ZT] 16). Error bars represent the standard deviation based on 3 biological replicates with 3 technical replicates each.

To test whether *SOC1*, which we found to be differentially expressed in response to dexamethasone application or in *hua2-4 tps1-2 GVG::TPS1*, is a major target of *HUA2* in the regulation of flowering time in *tps1-2 GVG::TPS1* we constructed the *soc1-2 hua2-4 tps1-2 GVG::TPS1* triple mutant. We observed that the triple mutant flowered only moderately later than the *hua2-4 tps1-2 GVG::TPS1* double mutant (Fig. 4, A and B; Supplementary Table S4, experiment 3). This indicates that even though *SOC1* is significantly induced in our RNA-seq experiment in *hua2-4 tps1-2 GVG::TPS1* (Fig. 3D; Supplementary Table S8) and in RT-qPCR experiments (Fig. 4C), *SOC1* is largely dispensable for the induction of flowering *in tps1-2 GVG::TPS1* by loss of *hua2*.

SOC1 is known to act partially upstream of the flowering time integrator gene and florigen FT. We, therefore, decided to test if induction of flowering in *tps1-2 GVG::TPS1* by *hua2-4* required functional *FT*. Interestingly, mutation of *FT* completely abolished the effect of *hua2-4* on flowering of *tps1-2 GVG::TPS1* and the *ft-10 hua2-4 tps1-2 GVG::TPS1* triple mutant failed to flower even after 4 mo of growth in inductive long-day conditions (Fig. 4, D and E; Supplementary Table S4, experiment 3). In line with this observation, we detected increased expression of *FT* at the end of the LD (ZT 16) in the *hua2-4 tps1-2 GVG::TPS1* double mutant when compared with *tps1-2 GVG::TPS1* (Fig. 4F). It is interesting to note that *FT* expression was barely detectable at ZT 4 according to our RNA-seq analysis (Supplementary Fig. S3A), which is in agreement with the diurnal expression pattern reported for *FT* (Kobayashi et al. 1999). Taken together, our genetic and molecular analyses indicate that *hua2-4* induces flowering of *tps1-2 GVG::TPS1* in part through activation of *FT*, with minor contributions of the upstream regulators *SOC1*.

### Loss of *FLC* induces flowering in *tps1-2 GVG::TPS1*

*HUA2* has previously been reported to regulate flowering at least in part by regulating the expression of floral repressors of the MADS-domain transcription factor family, including *FLC* and *FLOWERING LOCUS M* (Doyle et al. 2005). To test if *hua2-4* induces flowering in *tps-2 GVG::TPS1* through these repressors we constructed the *flc-3 hua2-4 tps1-2 GVG::TPS1* triple mutant. We found that this triple mutant flowered moderately earlier than *hua2-4 tps1-2 GVG::TPS1* (Fig. 4, G and H; Supplementary Table S4, experiment 3). In agreement with these findings, RT-qPCR analysis failed to detect *FLC* expression in the *hua2-4 tps1-2 GVG::TPS1* mutant, whereas *FLC* expression was readily detectable by RT-qPCR in *tps1-2 GVG::TPS1* (Fig. 4I).

**Figure 5.**
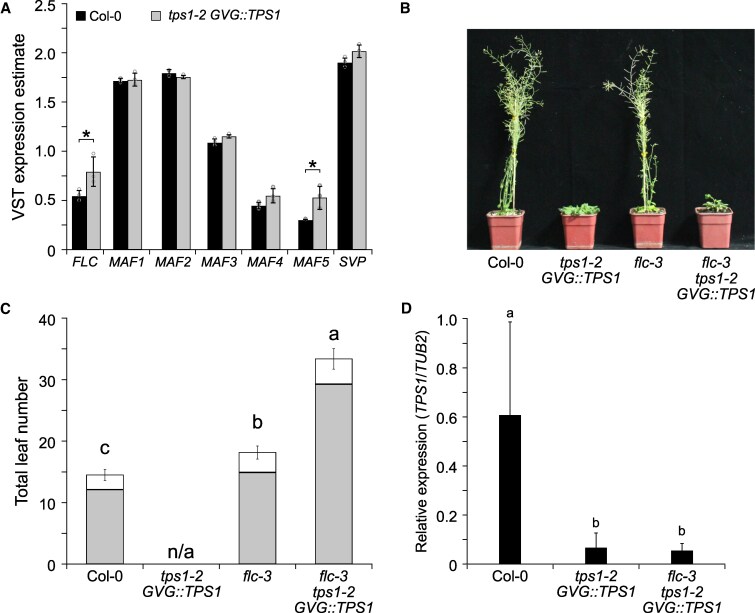
Loss of *FLC* rescues the non-flowering phenotype of *tps1-2 GVG::TPS1*. **A)** Variance stabilizing transformation (VST) expression estimates for MCM1, AGAMOUS, DEFICIENS, and SRF (MADS)-box floral repressors in 18-d-old plants. RNA-seq expression data retrieved from Zacharaki et al. (2022). Columns indicate mean VST expression estimates as implemented in DEseq2 calculated from 3 individual biological replicates per genotype. Col-0: black; *tps1-2 GVG::TPS1*: gray. Circles indicate expression estimates for individual biological replicates. Asterisks indicate differential gene expression with a statistical significance of *Padj*  *<*  *0.01* based on 3 biological replicates per genotype. **B**, **C)** Phenotypes **(B)** and total leaf number **(C)** of Col-0, *tps1-2 GVG::TPS1*, *flc-3*, and *flc-3 tps1-2 GVG::TPS1* double mutant*. GVG::TPS1* designates a dexamethasone-inducible *TPS1* transgene present in the genotype. Flowering time was scored as total leaf number (rosette [gray] and cauline leaves [white]) after bolting. Error bars represent the standard deviation of the total leaf number based on 20 individuals per genotype (Supplementary Table S4). ANOVA Tukey's multiple comparisons test was applied, and letters represent the statistical differences among genotypes (*P*  *<*  *0.001*). **D)** Expression of *TPS1* in col-0, *tps1-2 GVG::TPS1*, and *flc-3 tps1-2 GVG::TPS1*, in 28-d-old LD-grown plants. Samples were taken at zeitgeber (ZT) 4. Error bars represent the standard deviation based on 3 biological replicates with 3 technical replicates each. ANOVA Tukey's multiple comparisons test was applied, and letters represent the statistical differences among genotypes (*P*  *<*  *0.001*). LD, long-day.

**Figure 6.**
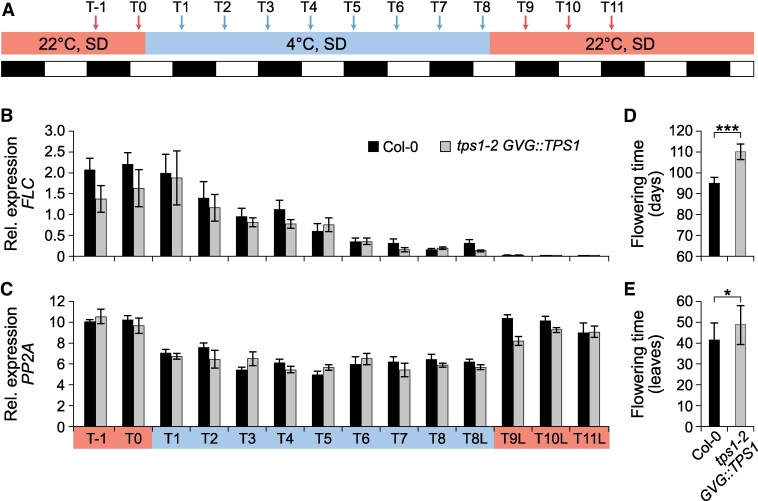
Vernalization induces flowering in *tps1-2 GVG::TPS1*. **A)** Experimental setup. Col-0 and *tps1-2 GVG::TPS1* plants were grown on soil under short days (SD) at 22 °C for 24 d, before being shifted to 4 °C for 8 wk for vernalization, after which plants were returned to 22 °C till flowering. *GVG::TPS1* designates a dexamethasone-inducible *TPS1* transgene present in the genotype. Samples were taken weekly for RT-qPCR analyses as indicated (arrows), starting 1 wk before the shift to 4 °C. **B**, **C)** RT-qPCR expression of *FLOWERING LOCUS C* (*FLC*) **(B)** and *SERINE/THREONINE PROTEIN PHOSPHATASE* (*PP2A*) **(C)** in Col-0 and *tps1-2 GVG::TPS1*. RNA for time points T1 to T7 was extracted from whole plants, while RNA for samples T9, T10, and T11 was isolated from leaves (L). For time point 8, RNA was extracted from both whole plants (T8) and leaves (T8L). Error bars show the standard deviation of 6 biological replicates (*n* = 6) for each time point. **D**, **E)** Flowering time of Col-0 (*n* = 11) and *tps1-2 GVG::TPS1* (*n* = 22) in days to flower **(D)** and total leaf number **(E)**. Asterisks indicate statistical significance according to a 2-tailed Student's *t*-test assuming unequal variance (*: *P*  *<*  *0.05*; *** *P*  *<*  *0.001*).

Furthermore, we found that the expression of *FLC* was significantly upregulated in 18-d-old *tps1-2 GVG::TPS1* seedlings when compared with Col-0 in publicly available RNA-seq data (Zacharaki et al. 2022) (Fig. 5A). This prompted us to test loss off *FLC* on its own might be sufficient to suppress the non-flowering phenotype of *tps1-2 GVG::TPS1*. Indeed, we observed that *flc-*3 alone is capable of inducing flowering in the otherwise non-flowering *tps1-2 GVG::TPS1* mutant background, even though the *flc-3 tps1-2 GVG::TPS1* double mutant flowered significantly later than wild-type and *flc-3* (Fig. 5, B and C; Supplementary Table S4, experiment 4). Importantly, we observed comparable levels of *TPS1* expression in *flc-3 tps1-2 GVG::TPS* and *tps1-2 GVG::TPS* (Fig. 5D), indicating that loss of *FLC* did not result in an activation of the *GVG::TPS1* transgene. The finding that loss of *FLC* rescued flowering in *tps1-2 GVG::TPS1* was surprising given that previous seed vernalization experiments had no such effect (van Dijken et al. 2004). However, in our conditions, vernalization of short-day-grown seedlings for 8 wk (Fig. 6A) resulted in strong and stable downregulation of *FLC* in both Col-0 and *tps1-2 GVG::TPS1* (Fig. 6, B and C) and flowering upon return to warm conditions (Fig. 6, D and E). These findings suggest that the failure of *tps1-2 GVG::TPS1* to flower could in part be due to *FLC*, possibly in conjunction with other MADS-box repressors such as *MADS AFFECTING FLOWERING 5* (*MAF5*), the expression of which was also elevated in *tps1-2 GVG::TPS1* (Fig. 5A). In contrast, expression of *HUA2* was not changed in *tps1-2 GVG::TPS1* when compared with Col-0 according to publicly available RNA-seq data (Supplementary Fig. S4).

### *hua2-4* attenuates carbon starvation responses

**Figure 7.**
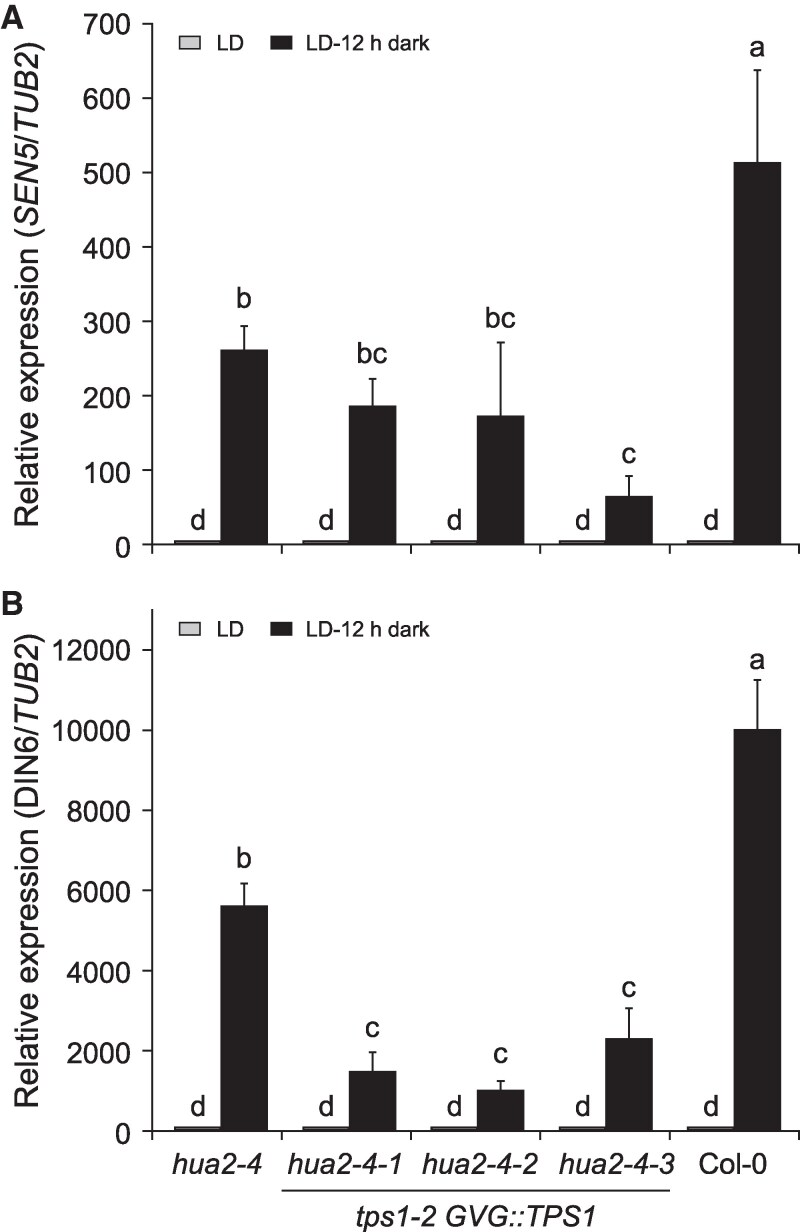
Expression of SnRK1 target genes *SEN5* and *DIN6* in *hua2-4* and *hua2-4 tps1-2 GVG::TPS1* double mutant. **A**, **B)** Induction of *SEN5*  **(A)** and *DIN6*  **(B)** in response to extended night is attenuated in *hua2-4* single mutant and 3 independent lines of the *hua2-4 tps1-2 GVG::TPS1* double mutant. *GVG::TPS1* designates a dexamethasone-inducible *TPS1* transgene present in the genotype. Plants were grown for 14 d in LD (gray) before being exposed to a single extended night (12 h additional darkness; black). LD, long days. Expression was determined by RT-qPCR using 3 biological replicates with 3 technical repetitions each and normalized to *TUBULIN BETA CHAIN 2* (*TUB2*). Error bars represent the standard deviation. ANOVA Tukey's multiple comparisons test was applied, and letters represent the statistical differences among genotypes (*P*  *<*  *0.001*).

The above data indicate that mutations in *HUA2* bypass the requirement for *TPS1* to induce flowering by reducing expression of MADS-box floral repressors and ultimately inducing floral integrator genes such as *FT* and *SOC1*. However, carbohydrate signaling has been shown to also indirectly regulate phase transitions, including flowering, in *A. thaliana* (Corbesier et al. 1998; Gibson 2005; Xing et al. 2015; Wang et al. 2020). In part, this response is mediated by SnRK1, which in response to stress conditions such as extended darkness phosphorylates a range of proteins, including several C- and S1-class bZIP transcription factors. Activation of these transcription factors by SnRK1 induces expression of stress response genes, including *SENESCENCE5* (*SEN5*) and *DARK INDUCED6*/*ASPARAGINE SYNTHASE1* (*DIN6*/*ASN1*), which can be used as a proxy for SnRK1 activity (Delatte et al. 2011; Dietrich et al. 2011; Mair et al. 2015). To test if loss of *HUA2* might affect flowering also more indirectly by modulating cellular energy responses, we analyzed the expression of *SEN5* and *DIN6*. Interestingly, we found that induction of *SEN5* and *DIN6* in response to extended night was strongly attenuated in *hua2-4* (Fig. 7, A and B) similar to what we had previously observed in mutants affected in SnRK1 subunits (Zacharaki et al. 2022). Induction of *SEN5* and *DIN6* in *hua2-4 tps1-2 GVG::TPS1* in response to an extended night was further reduced to approximately 20% to 30% of that observed in Col-0 wild-type (Fig. 7, A and B). However, this degree of downregulation is comparable to that observed in *tps1-2 GVG::TPS1* control plant (Supplementary Fig. S5), suggesting that *hua2-4* and *tps1-2* are not additive. However, it remains evident that *hua2-4* itself attenuates *SEN5* and *DIN6* induction in response to carbon starvation, which is in line with the results from our transcriptome-wide analyses (Fig. 3).

## Discussion

Arabidopsis *HUA2* has been reported to play a crucial role in various aspects of plant growth and development. *HUA2* was initially identified as an enhancer of the *AGAMOUS* (*AG*) allele *ag-4* (Chen and Meyerowitz 1999). Later, *HUA2* was found to also play a role as a repressor of flowering (Doyle et al. 2005; Wang et al. 2007). At the molecular level, *HUA2* has been suggested to function as a putative transcription factor but has also been implicated in RNA processing (Cheng et al. 2003). *HUA2* is expressed throughout the whole plant growth period (Chen and Meyerowitz 1999), indicating the importance and widespread effects on plant growth. Here, our study showed that loss of *HUA2* can partially restore flowering in *tps1-2 GVG::TPS1*.

It is interesting to note that in our EMS suppressor screen, we did not identify mutations in any of the *HUA2-like* genes, *HULK1, HULK2,* and *HUL3*, present in *A. thaliana* (Jali et al. 2014). One possible explanation is that our genetic screen might not have been saturated or that *HUA2*-like genes were missed due to the relatively low sequencing coverage. However, we believe this to be rather unlikely given that our approach has recovered multiple alleles in *HUA2* (this study) as well as 2 SnRK1 subunits (Zacharaki et al. 2022). Furthermore, flowering time is unaffected in the *hua2-like* single mutants, and *hulk2 hulk3* double mutants have been shown to be late flowering (Jali et al. 2014). Thus, it seems unlikely that mutation in any of the *HUA2-like* genes would suppress the non-flowering phenotype of *tps1-2 GVG::TPS1*.

*HUA2* has been reported to exert its function in part by regulating the expression of MADS-box transcription factors (Doyle et al. 2005), named after *MINICHROMOSOME MAINTENANCE 1* (*MCM1*) in yeast, *AGAMOUS* (*AG*) in Arabidopsis, *DEFICIENS* (*DEF*) in Antirrhinum, and serum response factor (SRF) in humans. MADS-BOX domain transcription factors contribute to all major aspects of the life of land plants, such as female gametophyte development, floral organ identity, seed development, and flowering time control (Portereiko et al. 2006; Colombo et al. 2008; Koo et al. 2010; Lee et al. 2013; Posé et al. 2013). In this context, it is interesting to note that our transcriptome and genetic analysis identified several MADS-box transcription factors to be misregulated in *tps1-2 GVG::TPS1*. In particular, the well-known floral repressors *FLC* and *MADS AFFECTING FLOWERING5* (*MAF5*) were found to be induced in *tps1-2 GVG::TPS1* compared with Col-0 (Fig. 5A). Moreover, loss of *FLC* was sufficient to induce flowering in *tps1-2 GVG::TPS1* (Fig. 5, B and C), suggesting that these floral repressors are partially responsible for the non-flowering phenotype of *tps1-2 GVG::TPS1*. Our transcriptome analyses further identified 2 MADS-box transcription factors, *SOC1* and *AGL24*, both known to promote flowering in Arabidopsis, to be upregulated in *hua2-4 tps1-2 GVG::TPS1*.

The molecular mechanism by which *HUA2* regulates the expression of these MADS-box flowering time regulators is currently unclear. However, since HUA2 localizes to the nucleus, it seems possible that HUA2 is directly involved in regulating the expression of these genes. For example, *HUA2* could (directly) promote the expression of *FLC*, which has previously been shown to directly bind to and repress the expression of *FT* and *SOC1* (Chen and Meyerowitz 1999; Doyle et al. 2005; Deng et al. 2011). In such a scenario, the increased expression of *FT*, *SOC1*, and *AGL24* in *hua2-4 tps1-2 GVG::TPS1* would be the result of reduced expression of floral repressors such as *FLC* and *MAF5.* However, the regulation of flowering is a very complex process full of intricate feedback loops, and *HUA2* might regulate *SOC1* and *AGL24* directly rather than indirectly. In this context, it is interesting to note that a nonfunctional *hua2* allele may compensate for the loss of *FLC* in *Ler* accession (Lemus et al. 2023). Alternatively, HUA2 might affect the expression of these important flowering time genes through interaction with RNA Pol-II via its CID domain, which is affected by the *hua2-13* alleles (R902C). Interestingly, polymorphisms resulting in amino acid substitutions in natural accessions of *A. thaliana* have been reported for R902 and A983, but not for P455 (The 1001 Genomes Consortium 2016). Even though the molecular mechanisms underlying *HUA2* function remain elusive, our results confirm *HUA2* as a central regulator of flowering time in Arabidopsis.

We have previously identified mutations in 2 subunits of SNF1-related kinase 1 (SnRK1), *KIN10* and *SNF4*, that partially restore flowering and seed set in *tps1-2 GVG::TPS1* (Zacharaki et al. 2022). The identification of these suppressor mutations was in line with the role of SnRK1 as a downstream regulator of the T6P pathway and other stresses (Baena-González and Lunn 2020; Avidan et al. 2023; Bortlik et al. 2024). Antagonizing SnRK1 in the regulation of energy homeostasis in plants is target of rapamycin (TOR), the activity of which is inhibited under energy-limiting conditions (Baena-González and Hanson 2017; Belda-Palazón et al. 2022). How exactly *HUA2* modulates carbon responses in Arabidopsis remains to be established. It is well-known that T6P signaling through SnRK1 affects processes such as carbon starvation response, germination, flowering, and senescence in opposition to the TOR (target of rapamycin) pathway (Figueroa and Lunn 2016; Baena-González and Lunn 2020). The regulatory network controlling this central metabolic hub is still not fully understood, and additional players are constantly added. For example, it has recently been shown that class II TPS proteins are important negative regulators of SnRK1 (Van Leene et al. 2022).

Regarding a possible role of *HUA2* in integrating carbon responses, it is worth noting that flavonoid-related genes (GO:0009812) were downregulated in *tps1-2 GVG::TPS1* in response to dexamethasone application and the *hua2* mutant (Fig. 3B). This is interesting as HUA2 is known to promote anthocyanin accumulation (Ilk et al. 2015), whereas SnRK1 has been shown to repress sucrose-induced anthocyanin production (Li et al. 2014; Meng et al. 2018; Broucke et al. 2023 ). Thus, *HUA2* might constitute an important hub in coordinating metabolic responses. However, as expression of SnRK1 subunits is not affected in *hua2-4 tps1-2 GVG::TPS1* when compared with *tps1-2 GVG::TPS1* (Supplementary Fig. S6), such a role would likely be indirect.

It is noteworthy that in our experimental conditions, the expression of *SEN5* and *DIN6* in response to an extended night is also significantly attenuated in *tps1-2 GVG::TPS1* (Supplementary Fig. S5). This might seem counterintuitive at first as trehalose 6-phosphate signaling has been reported to antagonize SnRK1 and thus, one would expect these genes to be induced in response to extended darkness. However, this notion is based, among others, on results from transient LUC reporter assays in hypomorphic *tps1* mutants (Frank et al. 2018). To the best of our knowledge, the expression of the endogenous SnRK1 target genes in response to extended night-induced starvation in *tps1* mutants has not yet been investigated. Our findings thus suggest that, although the TPS1/T6P pathway is generally suppressing the SnRK1 activity when sugar levels drop, the plant still requires a minimal amount of TPS1 expression under extended nutrient/carbon starvation. Furthermore, *tps1-2 GVG::TPS1* plants are already in energy-saving mode since SnRK1 is de-repressed. Thus, putting this mutant under additional extended night-induced carbon starvation stress could activate other conservatory mechanisms to prevent over-depletion of carbon, especially during the night when plants consume sugars to grow and sustain essential energy-demanding processes. This mechanism might be relieved when plants are returned to light conditions and energy is made available via photosynthesis. This mechanism could also explain the observed higher levels of *DIN6* and *SEN5* expression during the daylight period before the extended night.

Clearly, understanding the interplay between energy metabolism, in particular SnRK1, TOR, and T6P signaling, and plant growth and development is of utmost importance for developing plants capable of withstanding future challenges. The suppressor mutants generated in the *tps1-2 GVG::TPS1* background comprise an important resource in our hunt for additional factors that, like *HUA2*, link energy metabolism to plant development.

## Materials and methods

### Plant materials and growth conditions

All T-DNA insertion mutants and transgenic lines used in this work are in the Col-0 background. The *tps1-2 GVG::TPS1* line used in this work is referred to as ind-TPS1 #201 in the original publication (van Dijken et al. 2004). The *hua2-4* (SALK_032281C) was obtained from NASC and the presence of the T-DNA insertion was confirmed by PCR. *ft-10* (GABI-Kat: 290E08) was provided by Dr. Yi Zhang, Southern University of Science and Technology, *flc-3* (Michaels and Amasino 1999) by Dr. Liangyu Liu, Capital Normal University, and *soc1-2* (Lee et al. 2000) by Dr. Jie Luo, Chinese Academy of Sciences. *tps1-2 GVG::TPS1 hua2-4* plants were generated by crossing and double homozygous mutants were identified by phenotyping and genotyping of F2 individuals. Higher order mutants were obtained by crossing *soc1-2*, *flc-3*, and *ft-10* mutants with the *tps1-2 GVG::TPS1 hua2-4* double mutant and homozygous triple mutants were identified in the F2 and F3 generation. All mutant genotypes were confirmed by PCR, see Supplementary Table S10 for details. Plants were planted in nutrient soil with a normal water supply and grown under LD with a photoperiod of 16 h light at 22 °C and 8 h darkness at 20 °C. Flowering time was determined by counting the total number of leaves (rosette and cauline) derived from the shoot apical meristem and the number of days from germination to bolting (DTFs; inflorescence length, 0.5 cm) (Ponnu et al. 2020). For vernalization, seeds were stratified at 4 °C for 48 h and sown on soil. Plants were grown in Sd (approx. 150 *μ*mol m^−2^ s^−1^) at 22 °C for 24 d before being vernalized at 4 °C for 56 d in Sd (approx. 50 *μ*mol m^−2^ s^−1^), after which plants were returned to Sd (approx. 150 *μ*mol m^−2^ s^−1^) at 22 °C for an additional 44 d until all plants had started flowering.

### Dexamethasone treatment of *tps1-2 GVG:TPS1* mutant

For RNA-Seq and crossings, *tps1-2 GVG:TPS1* mutant plants were grown on soil. Starting 10 d after germination, plants were sprayed with a solution containing 1 *μ*m dexamethasone (Sigma) and 0.02% Tween-20 (Sigma) every 2nd d. Treatments were continued until plants were either harvested for RNA-seq 21 d after germination or until after flowering for crossings.

### Genome sequencing and analysis

Young leaves were used for DNA extraction for sequencing using the NovaSeq 6000 Sequencing platform (Novogene). Adapters and low-quality sequences of raw reads were trimmed using Trimmomatic (Bolger et al. 2014), and the clean reads were mapped to the reference genome of Col-0 using BWA-MEM (v0.7.15) (Cingolani et al. 2012). SNP calling was performed using Genome Analysis Toolkit 4 (GATK4; https://gatk.broadinstitute.org/hc/en-us) with default parameters. Variants were annotated using snpEff 4.3 (Li and Durbin 2009) based on TAIR 10 annotation. Next, we identified the protein-coding genes with multiple non-redundant mutations and found 3 mutant lines harboring unique non-synonymous mutations in the *HUA2* gene. The method was inspired by our previous study that multiple EMS-induced mutants with unique mutation sites in the coding regions of SnRK1 alpha subunit rescued the non-flowering phenotype of *tps1* (Zacharaki et al. 2022).

### Gene expression analysis by RNA-Seq

For RNA-seq analyses, plants were grown on soil for 3 wk in LD conditions. Leaves from 21-d-old Arabidopsis (*Arabidopsis thaliana*) were collected, immediately snap-frozen and stored at −80 °C. Total RNA was extracted using RNAprep Pure Plant Plus Kit (Tiangen, China, DP441). RNA integrity was assessed using the RNA Nano 6000 Assay Kit on the Bioanalyzer 2100 system (Agilent Technologies, CA, USA). RNA-seq libraries were generated with 3 independent biological replicates and sequenced on the Illumina NovaSeq platform by Annoroad Gene Technology. The raw RNA-seq reads were quality trimmed by Trimmomatic (v 0.11.9) (Bolger et al. 2014). The qualified reads were mapped to TAIR10 version genome guided by gene annotation model using HISAT2 (v2.1.0) (Kim et al. 2015). The expression level for each gene was determined by StringTie (v1.3.4) (Pertea et al. 2016). Differentially expressed genes (DEGs) were identified using Cuffdiff 2 using default settings with q-value (adjusted *P*-value) < 0.05 (Trapnell et al. 2012). The public RNA-seq datasets PRJNA471625 (Fu et al. 2021) for Col-0 plants under glucose depletion and glucose recovery and PRJNA430725 (Pedrotti et al. 2018) for Col-0 and SnRK1 knockdown plants were processed using the same procedures.

### RNA isolation and RT-qPCR data analysis

Total RNA was extracted from Arabidopsis seedlings using the RNA Isolation Kit (Tiangen, China, DP441) according to the manufacturer's instructions. cDNA was synthesized from 3 *µ*g total RNA in a 10 *µ*L reaction volume using the RevertAid Premium First Strand cDNA Synthesis Kit (Fermentas, Thermo Fisher Scientific, Rochester, NY). RT-qPCR was performed using TB Green Premix Ex Taq II (Takara, Dalian, China). Relative gene expression was calculated using the 2−ΔΔCt method. All analyses were repeated 3 times. The primers used for RT-qPCR are listed in Supplementary Table S10. For the vernalization experiment, RNA was extracted from seedlings and leaves using the RNeasy Plant kit from Qiagen according to the manufacturer's instructions. cDNA was synthezised from 1 *μ*g of total RNA using the RevertAid First Strand cDNA Synthesis Kit (Thermo Fisher Scientific) after which RT-qPCR was performed using SYBRgreen (Roche). Relative gene expression was calculated as described above.

### Accession numbers

Identifiers of key genes used in this study: *TPS1* (At1g78580), *HUA2* (AT5G23150), *SOC1* (AT2G45660), *FLC* (AT5G10140), and *FT* (AT1G65480). RNA-seq data generated in this study have been deposited with NCBI under the BioProject PRJNA1005425.

## Supplementary Material

kiaf225_Supplementary_Data

## Data Availability

RNA-sequencing data generated in this study is available from the NCBI BioProject PRJNA1005425. Other data that support the findings of this study are available within the figure and Supplementary materials or are available from the corresponding authors upon request.

## Supplementary data

The following materials are available in the online version of this article.

**Supplementary Figure S1.**  *TPS1* expression in *hua2-4 tps1-2 GVG::TPS1*.

**Supplementary Figure S2.** Relative expression of *HUA2* in *tps1-2 GVG::TPS1* treated with dexamethasone or untreated.

**Supplementary Figure S3.** Relative expression of important floral regulators.

**Supplementary Figure S4.** VST expression estimates for *HUA2* in 18-d-old plants.

**Supplementary Figure S5.** Expression of SnRK1 target genes *SEN5* and *DIN6* in *tps1-2 GVG::TPS1*.

**Supplementary Figure S6.** Relative expression of SnRK1 subunits.

**Supplementary Table S1.** Number of SNPs identified in individual suppressor mutants.

**Supplementary Table S2.** Number of SNPs identified in EMS suppressor lines carrying mutations in *HUA2.*

**Supplementary Table S3.** EMS suppressor lines bearing non-synonymous mutations in *HUA2*.

**Supplementary Table S4.** Flowering time and leaf number of different genotypes.

**Supplementary Table S5.** GO analysis of 392 genes downregulated in *tps1-2 GVG::TPS1* in response to dexamethasone application and in *hua2-4*.

**Supplementary Table S6.** RNA-seq expression estimates and statistical analysis of DEGs of 21-d-old *tps1-2 GVG::TPS1* (±DEX) and *hua2-4 tps1-2 GVG::TPS1* (-DEX) plants calculated using Cuffdiff 2.

**Supplementary Table S7.** GO analysis of 237 genes induced in *tps1-2 GVG::TPS1* in response to dexamethasone application and in *hua2-4*.

**Supplementary Table S8.** Expression of flowering time genes in *hua2-4 tps1-2 GVG::TPS1* and *tps1-2 GVG::TPS1*.

**Supplementary Table S9.** List and GO analysis of 700 known SnRK1 and TOR target genes regulated by *hua2-4* and dexamethasone application in *tps1-2 GVG::TPS1*.

**Supplementary Table S10.** List of oligonucleotides used in this study.

## Dive Curated Terms

The following phenotypic, genotypic, and functional terms are of significance to the work described in this paper:

HUA2 Gramene: AT5G23150
HUA2 Araport: AT5G23150

AGL24 Gramene: AT4G24540
AGL24 Araport: AT4G24540

AtTPS11 Gramene: AT2G18700
AtTPS11 Araport: AT2G18700

AtTPS4 Gramene: AT4G27550
AtTPS4 Araport: AT4G27550

SNF4 Gramene: AT1G09020
SNF4 Araport: AT1G09020

ASN1 Gramene: AT3G47340
ASN1 Araport: AT3G47340

AtTPS1 Gramene: AT1G78580
AtTPS1 Araport: AT1G78580

AtTPS2 Gramene: AT1G16980
AtTPS2 Araport: AT1G16980

AtTPS3 Gramene: AT1G17000
AtTPS3 Araport: AT1G17000

TSF Gramene: AT4G20370
TSF Araport: AT4G20370

PCR Gramene: Polymerase Chain Reaction
PCR Araport: Polymerase Chain Reaction

NCBI Gramene: https://www.ncbi.nlm.nih.gov
NCBI Araport: https://www.ncbi.nlm.nih.gov

TAIR Gramene: https://www.arabidopsis.org/
TAIR Araport: https://www.arabidopsis.org/

TUB2 Gramene: At5g62690
TUB2 Araport: At5g62690

KIN10 Gramene: AT3G01090
KIN10 Araport: AT3G01090
