# Supplementary material for: Mutations in the floral regulator gene HUA2 restore flowering to the Arabidopsis trehalose 6-phosphate synthase1 (tps1) mutant
Source: Plant Physiol. 2025 Jun 5;198(2):kiaf225. doi: 10.1093/plphys/kiaf225 (PMC12198769; doi:10.1093/plphys/kiaf225)
Supplement: kiaf225_Supplementary_Data [file kiaf225_supplementary_data.zip › Zeng_rev2_Supplementary_Material.pdf]

Mutations in the floral regulator gene HUA2 restore flowering to the Arabidopsis trehalose 6-phosphate synthase1 (tps1) mutant

Liping Zeng, Vasiliki Zacharaki, Sam W. van Es, Yanwei Wang, Markus Schmid

This document contains the following Supplementary Material

- Supplementary Figure S1
- Supplementary Figure S2
- Supplementary Figure S3
- Supplementary Figure S4
- Supplementary Figure S5
- Supplementary Figure S6
- Supplementary Table S1
- Supplementary Table S2
- Supplementary Table S3
- Supplementary Table S8
- Supplementary Table S10

Supplementary Table S4, Supplementary Table S5, Supplementary Table S6, Supplementary Table S7, and Supplementary Table S9 are provided as separate Excel files.

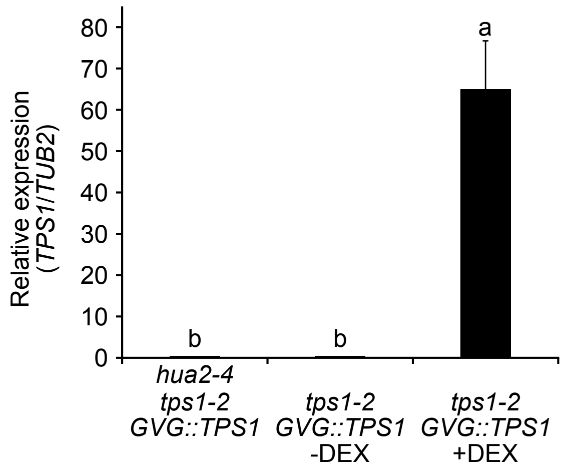

Supplementary Figure S1. *TPS1* expression in *hua2-4 tps1-2 GVG::TPS1*.

Expression of *TPS1* in *hua2-4 tps1-2 GVG::TPS1*, *tps1-2 GVG::TPS1 -DEX*, and *tps1-2 GVG::TPS1 +DEX* in 21-day-old plants at ZT relative to *TUB2*. Error bars represent the standard deviation based on three biological RNA-seq replicates. ANOVA Tukey's multiple comparisons test was applied, and letters represent the statistical differences among genotypes (P < 0.001).

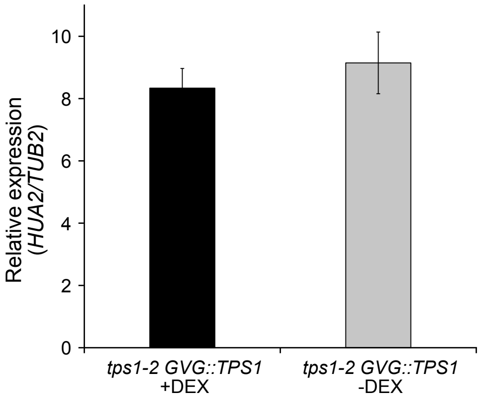

Supplementary Figure S2. Relative expression of *HUA2* in *tps1-2 GVG::TPS1* treated with dexamethasone or untreated.

Relative expression of *HUA2* in *tps1-2 GVG::TPS1* treated with dexamethasone (black) or untreated (grey) extracted from RNA-seq data. Error bars indicate SD based on three biological RNA-seq replicates. ANOVA Tukey's multiple comparisons test was applied. No statistically significant difference in *HUA2* expression was detected ($P > 0.05$).

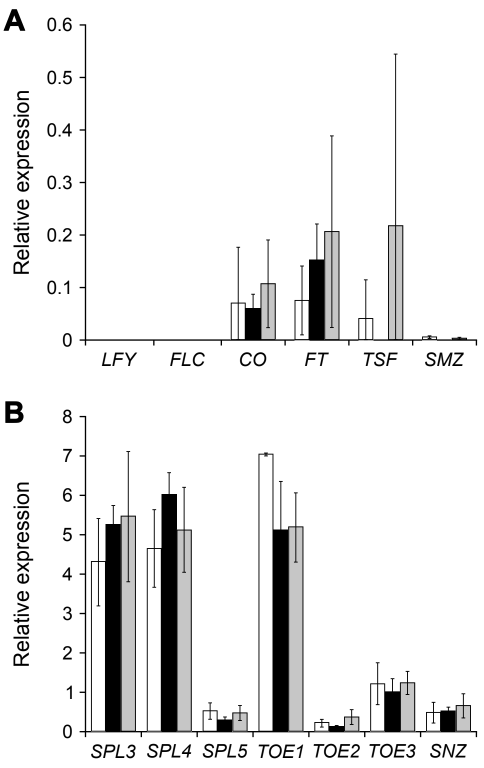

Supplementary Figure S3. Relative expression of important floral regulators.

Relative expression of important floral regulators in *tps1-2 GVG::TPS1* (white), *tps1-2 GVG::TPS1* treated with dexamethasone (black), and *hua2-4 tps1-2 GVG::TPS1* (grey) extracted from RNA-seq data normalized to *TUB2*. (**A**) floral regulators are expressed at low levels (**B**) or not differentially expressed. Error bars indicate SD based three biological RNA-seq replicates. ANOVA Tukey's multiple comparisons test was applied. No statistically significant differences in gene expression between genotypes were detected (P > 0.05).

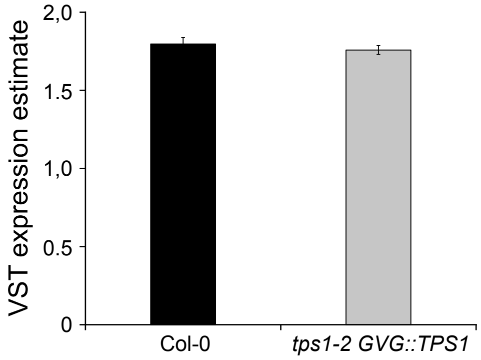

Supplementary Figure S4. VST expression estimates for *HUA2* in 18-day-old plants.
RNA-seq expression data based on three biological RNA-seq replicates retrieved from Zacharaki et al., 2022. Columns indicate mean VST expression estimates as implemented in DEseq2 calculated from three individual biological replicates per genotype. No statistically significant difference in *HUA2* expression was detected (P > 0.05).

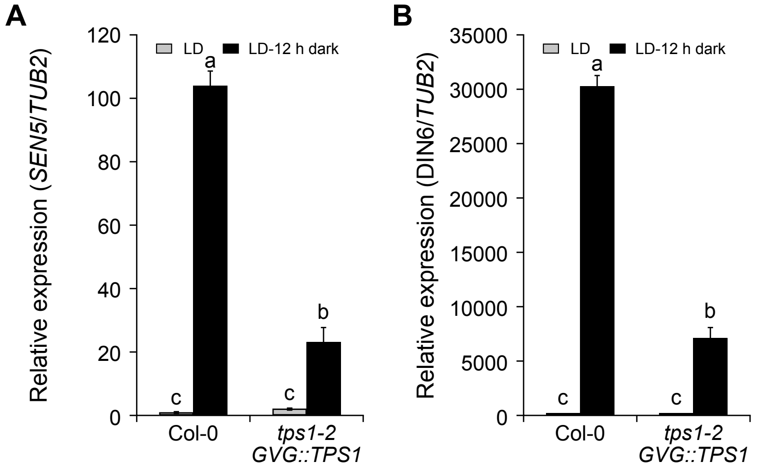

Supplementary Figure S5. Expression of SnRK1 target genes *SEN5* and *DIN6* in *tps1-2 GVG::TPS1*.

Induction of *SEN5* (**A**) and *DIN6* (**B**) in response to extended night is attenuated in *tps1-2 GVG::TPS1*. Plants were grown for 14 days in LD (grey) before being exposed to a single extended night (12h additional darkness; black). LD, long days. Error bars represent the standard deviation based on three biological RNA-seq replicates retrieved from Zacharaki et al., 2022. ANOVA Tukey's multiple comparisons test was applied, and letters represent the statistical differences among genotypes ($P < 0.001$).

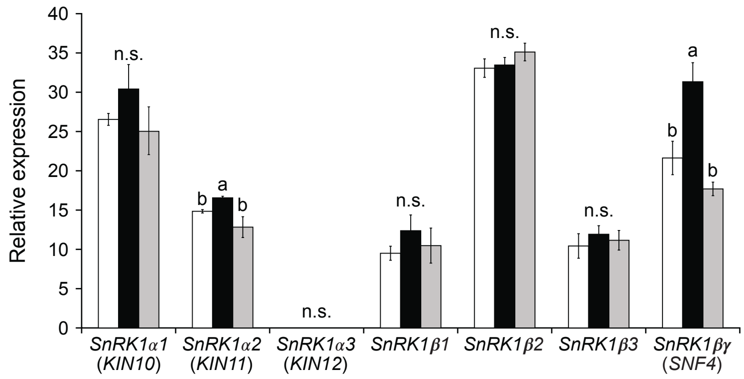

Supplementary Figure S6. Relative expression of SnRK1 subunits.

Relative expression of SnRK1 subunits in *tps1-2 GVG::TPS1* (white), *tps1-2 GVG::TPS1* treated with dexamethasone (black), and *hua2-4 tps1-2 GVG::TPS1* (grey). Error bars indicate SD based on three biological RNA-seq replicates. ANOVA Tukey's multiple comparisons test was applied. n.s. indicates non-significant differences.

Supplementary Table S1. Number of SNPs identified in individual suppressor mutants.

Nr	Line ID	SNPs	Publication
**1**	3-3-1	1242	Zacharaki et al., 2022
**2**	140-2-1	1179	Zacharaki et al., 2022
**3**	1-3-2	1008	Zacharaki et al., 2022
**4**	57-1-2	992	Zacharaki et al., 2022
**5**	228-1-2	946	Zacharaki et al., 2022
**6**	160-1	907	Zacharaki et al., 2022
**7**	75-2-1	904	Zacharaki et al., 2022
**8**	175-2-1	880	Zacharaki et al., 2022
**9**	233-14-1	880	Zacharaki et al., 2022
**10**	243-5-1	863	Zacharaki et al., 2022
**11**	92-2-1	855	Zacharaki et al., 2022
**12**	130-1-1	854	Zacharaki et al., 2022
**13**	8-1-1	853	Zacharaki et al., 2022
**14**	57-2-1	845	Zacharaki et al., 2022
**15**	79-5-2	842	Zacharaki et al., 2022
**16**	183-1-1	819	Zacharaki et al., 2022
**17**	255-5-1	816	Zacharaki et al., 2022
**18**	199-1-1	809	Zacharaki et al., 2022
**19**	72-1-2	790	Zacharaki et al., 2022
**20**	79-4-1	785	Zacharaki et al., 2022
**21**	144-1-1	781	Zacharaki et al., 2022
**22**	225-5-1	777	Zacharaki et al., 2022
**23**	144-2-1	774	Zacharaki et al., 2022
**24**	125-6-1	773	Zacharaki et al., 2022
**25**	58-6-2	757	Zacharaki et al., 2022
**26**	158-5-2	743	Zacharaki et al., 2022
**27**	103-5-1	714	Zacharaki et al., 2022
**28**	32-7-1	710	Zacharaki et al., 2022
**29**	48-2-2	710	Zacharaki et al., 2022
**30**	92-3-2	694	Zacharaki et al., 2022
**31**	54-4-1	693	Zacharaki et al., 2022
**32**	48-1-2	683	Zacharaki et al., 2022
**33**	170-1-1	678	Zacharaki et al., 2022
**34**	236-10-1	659	Zacharaki et al., 2022
**35**	91-1-21-2	653	Zacharaki et al., 2022
**36**	131-13-1	649	Zacharaki et al., 2022
**37**	101-4-2	645	Zacharaki et al., 2022
**38**	171-2-1	645	Zacharaki et al., 2022
**39**	171-1-1	637	Zacharaki et al., 2022
**40**	199-6-2	634	Zacharaki et al., 2022
**41**	184-1-2	620	Zacharaki et al., 2022
**42**	244-2-2	617	Zacharaki et al., 2022
**43**	155-3-1	613	Zacharaki et al., 2022
**44**	154-1-1	597	Zacharaki et al., 2022
**45**	103-2-2	595	Zacharaki et al., 2022
**46**	101-3-1	594	Zacharaki et al., 2022
**47**	155-2-1	542	Zacharaki et al., 2022
**48**	131-29-2	526	Zacharaki et al., 2022
**49**	225-1-1	519	Zacharaki et al., 2022
**50**	105-1-2	489	Zacharaki et al., 2022
**51**	163-5-2	481	Zacharaki et al., 2022
**52**	232-2-1	474	Zacharaki et al., 2022
**53**	196-1-2	453	Zacharaki et al., 2022
**54**	212-2-2	383	Zacharaki et al., 2022
**55**	180-3-1	343	Zacharaki et al., 2022
**56**	164-9-1	328	Zacharaki et al., 2022
**57**	180-1-1	282	Zacharaki et al., 2022
**58**	50-1-2	33	Zacharaki et al., 2022
**59**	292-2-1	31	Zacharaki et al., 2022
**60**	75-1-1	25	Zacharaki et al., 2022
**61**	233-13-1	24	Zacharaki et al., 2022
**62**	192-1-2	22	Zacharaki et al., 2022
**63**	292-1-1	21	Zacharaki et al., 2022
**64**	192-2-2	20	Zacharaki et al., 2022
**65**	230-2-2	17	Zacharaki et al., 2022
**66**	49-5-3	1523	this publication
**67**	132-1-2	1372	this publication
**68**	219-1-2	1100	this publication
**69**	91-1-21	980	this publication
**70**	106-2-1	731	this publication
**71**	232-2-2	716	this publication
**72**	292-1-2	74	this publication
**73**	34-8-1	74	this publication
**74**	192-1-1	64	this publication
**75**	50-1-1	82	this publication
**76**	230-2	63	this publication
**77**	278-1-1	62	this publication
**78**	30-34	941	this publication
**79**	107-2	1032	this publication
**80**	42-9	418	this publication
**81**	128-1	104	this publication
**82**	30-23	220	this publication
**83**	55-21	1035	this publication
**84**	55-6	905	this publication
**85**	55-15	371	this publication
**86**	11-7	833	this publication
**87**	77-3	1246	this publication
**88**	2-1	1240	this publication
**89**	160-1-b	916	this publication
**90**	161-1	685	this publication
**91**	271-1	1384	this publication
**92**	41-18	655	this publication

Supplementary Table S2. Number of SNPs identified in EMS suppressor lines carrying mutations in *HUA2*.

Line ID	DNA source	Number SNPs
8-1-1	Individual suppressor mutant	853
30-34	Individual suppressor mutant	941
57-2-1	Individual suppressor mutant	845
233-14-1	Individual suppressor mutant	880
164-9-1	Individual suppressor mutant	328

Supplementary Table S3. EMS suppressor lines bearing non-synonymous mutations in *HUA2*.

Gene isoform	Number of line(s) SNP is present	Line(s) ID	Chromosomal SNP position	Reference base	Alternative base	Feature	Codon position in gene	Length of CDS	Position of SNP in the CDS	Position of SNP in codon	Non-synonymous/ Synonymous	Reference amino acid	Alternative amino acid	Degeneracy
AT5G23150.1	3	8-1-1<br>30-34<br>57-2-1	7790473	G	A	CDS	4639	4179	2947	1	Nonsyn	A	T	Gcg
AT5G23150.1	1	233-14-1	7788166	C	T	CDS	2332	4179	1363	1	Nonsyn	P	S	Cca
AT5G23150.1	1	164-9-1	7790099	C	T	CDS	4265	4179	2704	1	Nonsyn	R	C	Cgt

Supplementary Table S8. Expression of flowering time genes in *hua2-4 tps1-2 GVG::TPS1* and *tps1-2 GVG::TPS1*.

Gene_ID	start	end	strand	*tps1-2 GVG::TPS1 hua2-4*			*tps1-2 GVG::TPS1*		
Replicate				1	2	3	1	2	3
AT4G24540(*AGL24*)	12670965	12674072	-	23,124781	25,085474	23,208811	11,087811	13,027147	10,491028
AT2G45660(*SOC1*)	18807538	18811047	-	8,265232	10,200581	12,231363	4,341604	4,784088	6,764344
AT5G61850(*LFY*)	24844295	24846933	+	0	0	0	0	0	0
AT5G10140(*FLC*)	3173497	3179448	-	0	0	0	0	0	0
AT5G15840(*CO*)	5171182	5172758	-	0,013072	0,140295	0,169611	0,194254	0	0,017146
AT1G65480(*FT*)	24331428	24333934	+	0,419172	0,094804	0,110622	0,11757	0,109185	0
AT4G20370(*TSF*)	11000771	11002996	-	0	0,059687	0,593648	0,125811	0	0
AT3G54990(*SMZ*)	20373718	20376522	-	0	0,002427	0,005711	0,00271	0,00165	0,009862
AT2G33810(*SPL3*)	14305001	14306072	+	4,384162	4,665609	7,363255	5,170671	4,712061	3,068198
AT1G53160(*SPL4*)	19806419	19807608	+	3,940252	5,441206	6,007432	5,306567	5,117388	3,515887
AT3G15270(*SPL5*)	5140365	5141348	-	0,289313	0,464	0,67997	0,533319	0,719163	0,33364
AT2G28550(*TOE1*)	12225842	12228543	-	4,908707	4,502523	6,183015	7,100536	7,014573	7,059748
AT5G60120(*TOE2*)	24207786	24211724	+	0,373802	0,578502	0,191514	0,318693	0,131952	0,211047
AT5G67180(*TOE3*)	26801949	26804249	-	1,491339	0,927218	1,287269	0,731689	1,146463	1,783746
AT2G39250(*SNZ*)	16388886	16391073	-	0,723291	0,935247	0,324612	0,66105	0,179	0,620404

Supplementary Table S10. List of oligonucleotides used in this study.

Purpose	*Target*	Sequence (5' => 3')
Genotyping *ft-10*	*ft-10*	AGGGTTGCTAGGACTTGGAACA
Genotyping *ft-10*	*ft-10*	CCCATTTGGACGTGAATGTAGACAC
Genotyping *ft-10*	*T-DNA* border	GGTGGAGAAGACCTCAGGAAC
Genotyping *hua2-4*	*hua2-4*	TCTATCAGAGCCACCTGCTTC
Genotyping *hua2-4*	*hua2-4*	TTACTCGGTCAGATTCCATGG
Genotyping *hua2-4*	*T-DNA* border	ATTTTGCCGATTTCGGAAC
Genotyping *soc1*	*soc1*	TGTGTGCAAGGGAAATTAACTAAAGAAGAAGAT
Genotyping *soc1*	*soc1*	TTAGTATGCCTCAGATAACGATCTATGGTAT
Genotyping *soc1*	*T-DNA* border	ATTTTGCCGATTTCGGAAC
Genotyping *flc*	*flc*	AGCCAAGAAGACCGAACTCA
Genotyping *flc*	*flc*	TTTGTCCAGCAGGTGACATC
Genotyping and sequencing EMS lines (233-14-1)	*hua2*	TGGAGCATGCTACCTCTCCT
Genotyping and sequencing EMS lines (233-14-1)	*hua2*	TCCTGCAACGTGCTTGTTAG
Genotyping and sequencing EMS lines (164-9-1)	*hua2*	TCCTGAAGTTGTGGCTTGAA
Genotyping and sequencing EMS lines (164-9-1)	*hua2*	TTCATGATCGACATCCTCCA
Genotyping and sequencing EMS lines (8-1-1, 30-34, 57-2-1)	*hua2*	TTTTTCTGCAGCAATGCAAC
Genotyping and sequencing EMS lines (8-1-1, 30-34, 57-2-1)	*hua2*	AGGTGGAGGTGAAAGTGGTG
Genotyping *tps1-2*	*tps1-2*	GACACTTGGTTTCTTGATATGTCCTG
Genotyping *tps1-2*	*tps1-2*	GCTGTCTTGGATACTGAACCAGT
Genotyping *tps1-2*	*tps1-2* transposon	GAGCGTCGGTCCCCACACTTCTATAC
RT-qPCR	*TUB2*	GAGCCTTACAACGCTACTCTGTCTGTC
RT-qPCR	*TUB2*	ACACCAGACATAGTAGCAGAAATCAAG
RT-qPCR	*UBI*	CACACTCCACTTGGTCTTGCGT
RT-qPCR	*UBI*	TGGTCTTTCCGGTGAGAGTCTTCA
RT-qPCR	*TPS1*	GAAACTCAAGACGTCCTTCACCAG
RT-qPCR	*TPS1*	TCTAGCATTGGTGCGAGTACGAC
RT-qPCR	*SOC1*	TTGAGCAGCTCAAGCAAAAGGA
RT-qPCR	*SOC1*	TCCCCACTTTTCAGAGAGCTTCTC
RT-qPCR	*FT*	CCCTGCTACAACTGGAACAAC
RT-qPCR	*FT*	CACCCTGGTGCATACACTG
RT-qPCR	*FLC*	AGCCAAGAAGACCGAACTCA
RT-qPCR	*FLC*	TTTGTCCAGCAGGTGACATC
RT-qPCR (vernalization experiment)	*FLC*	GAAGACCGAACTCATGTTGAAGCT
RT-qPCR (vernalization experiment)	*FLC*	GCTCCCACATGATGATTATTCTCC
RT-qPCR (vernalization experiment)	*PP2A*	ACTGCATCTAAAGACAGAGTTCC
RT-qPCR (vernalization experiment)	*PP2A*	CCAAGCATGGCCGTATCATGT
